# Trigeminal trophic syndrome presenting as Morgellons disease with a 2-year delay in diagnosis

**DOI:** 10.1016/j.jdcr.2025.11.028

**Published:** 2025-11-27

**Authors:** Mariana Chrispim Gimenez, Jay R. Patel, John DeAngelis, Francisco Tausk, Margaret R. Puelle

**Affiliations:** a University of Rochester School of Medicine and Dentistry, Rochester, New York; b Department of Dermatology, University of Rochester Medical Center, Rochester, New York; c Department of Emergency Medicine, University of Rochester Medical Center, Rochester, New York; d Department of Psychiatry, University of Rochester Medical Center, Rochester, New York

**Keywords:** mental nerve block, trigeminal trophic syndrome

## Introduction

Trigeminal trophic syndrome (TTS) is a rare etiology causing facial ulceration. Although its pathogenesis has not been fully understood, it usually, but not always, occurs secondary to insult to the trigeminal nerve, either central or peripheral.^1^, ^2^, ^3^ This condition manifests as persistent ulceration of the face and hypoesthesia and paresthesia along the trigeminal dermatome distribution.^1^ We report on a 33-year-old female patient who presented to the emergency department (ED) with concerns of left-sided facial pain in the setting of a skin lesion located on her left chin.

## Case report

A 33-year-old woman with a one-and-a-half-year history of a cutaneous left chin lesion presented to the ED at Strong Memorial Hospital due to an acute pain episode refractory to oral medications and impacting her ability to perform daily living activities. This patient had been followed by the psychodermatology clinic (which is staffed by both a dermatologist and psychiatrist) at the University of Rochester Medical Center for the prior 9 months for this lesion, which she reported exuded synthetic fibers, hard crystals, and microscopic unidentified black substances not visible upon examination, consistent with the phenomenon known as Morgellons disease. Medication trials for this condition included 23% lidocaine + 7% tetracaine cream, pregabalin, gabapentin, memantine, duloxetine, acetaminophen, corticosteroid ointments, and mupirocin ointment with limited symptom improvement. The patient was also taking 2400 mg of ibuprofen daily, with unsuccessful attempts to decrease this due to worsening pain despite counseling on the risks of daily nonsteroidal anti-inflammatory drug use. In the 2 weeks prior to her presentation to the ED, she had also been prescribed short courses of valproic acid, olanzapine, and lorazepam, without significant effect. Medical history was significant for remote history of a pituitary tumor status post resection, depression, anxiety, and attention-deficit/hyperactivity disorder, and trauma history was significant intimate partner violence in previous relationships and emotional abuse in childhood.

**Fig 1:**
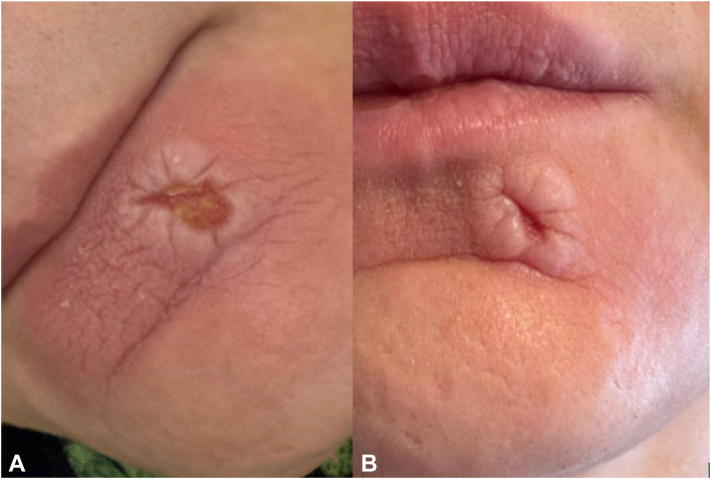
Chin lesion. **A,** Upon presentation to ED. **B,** Upon contacting psychodermatology clinic after initial nerve block.

Upon presentation to the ED, the patient was offered a mental nerve block for pain control, as the lesion was located within its distribution (see the mental nerve image by Volker^4^ and Fig 1). The mental nerve block was performed through the inner mucous membranes with 5 mL of bupivacaine 0.5% + epinephrine. After the intervention, the patient reported complete pain resolution and was admitted for observation. Following consultation with psychiatry she also received 1-time doses of pregabalin, droperidol, and IV valproic acid. She was discharged with a plan to follow up outpatient in the psychodermatology clinic.

**Fig 2:**
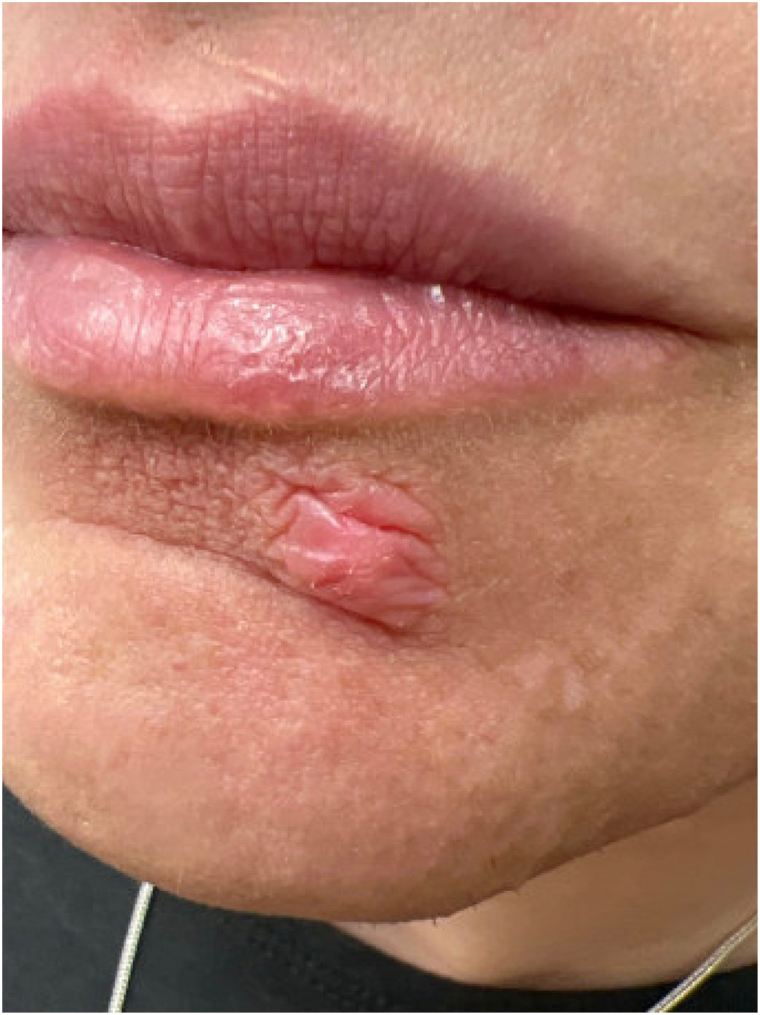
Chin lesion 7 months after initial mental nerve block.

Two days after discharge, the patient messaged the psychodermatology clinic reporting that her pain remained minimal and provided a picture showing significant improvement in localized inflammation (Fig 1). She has now been followed in the psychodermatology clinic for 10 months since the encounter at the ED. Her pain did recur, however, never as severe as it had been previously, and mental nerve blocks were repeated by her dermatologist in clinic 1 and 3 months after the initial procedure with ongoing positive effects and minimal reports of particles exuding from the lesion. On examination, the lesion has fully reepithelialized and decreased in size (Fig 2). The patient has now discontinued acetaminophen and ibuprofen and is only using pregabalin as needed for occasional pain exacerbations. She reports dramatically improved quality of life and resolution of functional impairments related to her facial pain or skin. Based on clinical presentation and improvement after the mental nerve block, the patient was ultimately diagnosed with TTS and continues to receive care for this condition.

## Discussion

TTS is an uncommon cause of facial ulceration associated with injury to the trigeminal nerve and/or its branches.^1^, ^2^, ^3^ The exact pathogenesis of this condition has yet to be elucidated, but it is associated with 2 typical presentations. In one, the patient experiences a known disturbance or injury to the trigeminal nerve. This condition often presents following procedures to manage the pain of trigeminal neuralgia, such as surgical ablation, alcohol injection, or severance of the sensory compartment of the trigeminal nerve, but it is also associated with noniatrogenic etiologies, including vascular insufficiencies and nervous system infections and tumors.^3^^,^^5^ At other times, as in this case, a self-inflicted ulceration develops within the distribution of the trigeminal nerve, likely due to hypoesthesia/paresthesia and resulting in manipulation and mutilation of tissue, without a clear preceding neurologic injury.^3^^,^^5^ There is debate regarding whether primary neuroinflammation is part of the syndrome or if inflammation is due solely to the physical trauma to the skin.^3^^,^^5^

Morgellons disease refers to the belief by patients that they and/or their surroundings are infested by inorganic materials, such as fibers, with reports of abnormal tactile sensations.^6^^,^^7^ Our patient reported inanimate materials present in her chin lesion, which could not be observed on examination. Notably, she sought care from a dermatologist as her subjective experience was of a problem with her skin. Her dramatic and sustained improvement, including reepithelization of the lesion, significant decrease in inflammation, and cessation of reports of inorganic materials exuding from the site, support the diagnosis of TTS, a neurologic disease process. Although the initial symptomatology fit the description of Morgellons disease, we believe it is more likely a feature of this patient’s presentation of TTS, rather than an isolated diagnosis.

Our case demonstrates the efficacy of mental nerve blocks in the treatment of TTS when localized to the V3 branch of the trigeminal nerve, the usefulness of this intervention as a continued outpatient procedure, and the importance of considering neurologic etiology for atypical dermatologic symptoms. The use of bedside nerve blocks has been documented for the treatment of scalp pruritus through occipital nerve blocks, also with good clinical outcomes.^8^ This case report introduces a new use to this procedure, with lasting results and feasible clinical management. Further research is warranted to elucidate the extended duration of analgesic effects of bupivacaine in these conditions.^8^

In summary, we present a case of TTS with confounding features of Morgellons disease with ultimate diagnosis after successful treatment with mental nerve blocks. This case report introduces a new intervention for the condition with effective therapeutic results.

## Conflicts of interest

None disclosed.
